# Attitudes Toward Social Media Versus Voting Among Adolescents and Youth in a Politicized Context: Chile Before and After the 2019 Social Uprising (2018–2022)

**DOI:** 10.3390/bs15101318

**Published:** 2025-09-26

**Authors:** Rodrigo Torres

**Affiliations:** Centro de Investigación en Ciencias Sociales y Juventud (CISJU), Universidad Católica Silva Henríquez, Santiago 8330311, Chile; rtorrest@ucsh.cl

**Keywords:** political participation, social media, non-conventional participation, voting, political attitudes, Chile’s 2019 October, political behavior

## Abstract

Following Chile’s October 2019 Social Uprising, social media increased as a key arena for youth political expression, leading us to investigate how adolescents (15–17) and young adults (18–21 and 22–24) transformed their attitudes toward social media as a more effective tool than voting to voice people’s demands. To this end, we analyzed nationally representative data from the 9th National Youth Survey (2018–2019, pre-Uprising) and the 10th National Youth Survey (2021–2022, post-Uprising), employing bivariate tests and multiple linear regressions to assess age-group differences and sociopolitical predictors: political interest, satisfaction with democracy, and political identification. Our findings indicate that, in the post-Social Uprising period, support for social media over voting increased across all cohorts. This increase was statistically significant, with the largest rise observed among adolescents. Moreover, young people with lower political interest and weaker political identification were more likely to value social media over voting, while those more satisfied with democracy also tended to perceive social media as an effective channel for voicing people’s demands. Taken together, these results underscore the transformative impact of sociopolitical crises on digital engagement patterns, particularly among less politicized youth, and highlight the importance of developmental and motivational distinctions when designing civic-education programs and online engagement strategies tailored to adolescents versus young adults.

## 1. Introduction

Over the past decade, social media have evolved from mere entertainment platforms into strategic arenas for political communication and mobilization. Recent research demonstrates that viral messages and campaigns on Facebook, X (formerly Twitter), Instagram, or TikTok can shape public opinion and even influence large-scale electoral outcomes, as observed in the Brexit referendum, the 2020 U.S. presidential election, and the 2024 European Parliament elections (Bossetta et al., 2023; Bossetta & Schmøkel, 2022; Daniel & Obholzer, 2025). This digital turn has redefined the repertoire of citizen participation in democracies: rallies and televised debates alone are no longer sufficient, as the “battle” for the attention of millions of users—who inform themselves, share, and debate online—shifts much political action into the continuous stream of digital content (Gupta & Chauhan, 2023).

Adolescents and young adults play a central role in this transformation. They interact with social media daily and use these platforms as more immediate, informal channels of expression and participation (Karimi & Fox, 2023; Oden & Porter, 2023). For many, “likes,” “shares,” and comments are not mere virtual gestures but symbolic and practical acts that enable the articulation of demands and critiques outside traditional institutional channels (Pontes et al., 2018). Moreover, this seamless transition from the playful to the political reflects growing distrust in formal institutions: digital platforms respond with greater immediacy and horizontality, functioning as new civic agoras themselves in which the meaning of citizenship is both constructed and contested.

The rise in social media has opened up non-conventional or non-institutional forms of participation, posing questions about their legitimacy and efficacy compared to classical democratic channels (Ruiz-Dodobara et al., 2025; Acheampong & Taden, 2024). In contexts of institutional disengagement and skepticism toward traditional representation mechanisms such as voting (Waeterloos et al., 2024), it becomes important to investigate which media young people perceive as most effective for channeling their social demands. Understanding how adolescents and young adults view these new forms of digital participation in relation to democracy is important for assessing the quality of contemporary political systems.

In Latin America, where social protests have been recurrent and high levels of inequality coexist with exponential growth in Internet access (Ruiz-Dodobara et al., 2025; Valenzuela et al., 2016; ITU, 2024), understanding the dynamics of youth digital participation and its valuation relative to voting is highly relevant. Accordingly, this study examines the case of Chile, a country marked by significant social mobilizations, political transformations, and one of the highest rates of Internet access in the region (ITU, 2024; Cadem-Subtel, 2025). In this context, analyzing how young people perceive the effectiveness of social media versus voting is relevant. Indeed, despite the burgeoning literature on digital participation, few studies have compared youth political behavior before and after disruptive sociopolitical events in highly politicized contexts (Eckstein et al., 2024; Scherman et al., 2021). Thus, the October 2019 Social Uprising—a nationwide wave of protests triggered by a fare increase on Santiago’s Metro system and fueled by broader demands regarding inequality in health and education and distrust in political institutions—together with the subsequent Constitutional Process (2021–2022) initiated in response to these mobilizations, provide a unique opportunity to examine how a social crisis and institutional reforms can reshape the ways in which adolescents and young adults value social media relative to voting.

To address this gap, the present study analyzes nationally representative data collected by Chile’s National Youth Survey in two periods: December 2018–April 2019 and December 2021–May 2022.

### 1.1. Social Media and Political Attitudes

The literature identifies three key attitudes that shape young people’s preference for social media over voting as a form of civic expression: political interest, satisfaction with democracy, and political identification (Lorenz-Spreen et al., 2023; Chan & Yi, 2024; Placek, 2023).

Political interest is defined as individuals’ deliberate attention to news, debates, and public affairs. Numerous studies have consistently shown that political interest is a strong predictor of both online and offline political participation (Lorenz-Spreen et al., 2023; Koivula et al., 2021; Moeller et al., 2018). Individuals with higher political interest tend to use social media for political purposes—consuming informative content, engaging in extended discussions, and commenting on posts—which in turn strengthens their civic commitment. Thus, political interest not only motivates voting but also guides media exposure and fosters engagement in new online spaces (Oden & Porter, 2023; Acheampong & Taden, 2024). These findings suggest that political interest may play a moderating role in the shift from traditional political engagement to digital platforms, influencing perceptions of social media as a legitimate channel for civic action. However, research in the United Kingdom has also found that those with low political interest often participate more actively in online discussions, indicating that social media can attract both highly engaged and more skeptical individuals (Bimber et al., 2014). In the Latin American context, Villagrán et al. (2023) observed a significant correlation between political interest and civic use of Facebook following the 2019 social uprisings in Chile and Ecuador. Specifically in Chile, recent studies report that civic use of social media positively impacts both internal and external political efficacy (Fierro et al., 2023). Yet, the specific link between political interest and the preference for social media over voting among Chilean youth—before and after the Social Uprising—remains unexamined in longitudinal research. This study seeks to fill that gap by evaluating the extent to which political interest predicts changes in youth perceptions of social media as a tool for civic expression.

Democratic satisfaction significantly influences perceptions of political efficacy and preferred modes of participation. In democracies with high levels of public appreciation, citizens tend to trust electoral processes more and view voting as a legitimate mechanism of influence. Conversely, when democracy is perceived as defective, citizens—especially young people—often turn to alternative forms of participation such as digital activism on social media (Acheampong & Taden, 2024; Lorenz-Spreen et al., 2023; Chan & Yi, 2024). Comparative studies indicate that social media’s impact on democratic satisfaction is context-dependent: in Europe, for instance, social media use correlates with higher democratic satisfaction in countries with strong institutions, whereas in contexts of democratic backsliding, this relationship reverses (Placek, 2023). Complementary research shows that in nations with high affective polarization, intensive social media use increases democratic engagement (e.g., voting and participation) but decreases satisfaction with democracy; in contrast, in low-polarization contexts, it enhances perceptions of democratic quality without significantly affecting political satisfaction (Chan & Yi, 2024). Moreover, the positive effect of social media on democracy intensifies in countries with higher internet penetration, underscoring digital access as a structural condition for robust civic participation (Acheampong & Taden, 2024). In the recent Chilean case, social media played a central role as spaces for expressing discontent and collective mobilization during the 2019 protests (Villagrán et al., 2023; Scherman & Rivera, 2021; Giacoman & Torres, 2021), confirming that in sociopolitical crises, these platforms become key channels for nonconventional political action.

Finally, the recent literature suggests that adolescents’ and young adults’ political identification is an important driver of how they evaluate social media relative to voting. Those with clear political affiliations tend to use platforms like Instagram or Twitter to amplify their messages and mobilize peers, without abandoning voting as their primary means of political expression (Oden & Porter, 2023; Maurissen & Claes, 2023). In Chile (2017–2019), studies show that youth who self-identify on the left or right used social media more extensively for political information (Espinoza Bianchini et al., 2022). Conversely, a growing segment of non-ideological or moderate youth—who do not identify with any party or ideology—are more likely to view social media as their principal avenue for voicing demands. For instance, in Belgium, citizens who had withdrawn from voting—a form of electoral defection—exhibited high levels of social media engagement as an alternative form of protest and expression (Waeterloos et al., 2024). In sum, whether young people do or do not identify politically shapes their perceptions: the highly politicized treat social media as an extension of their activism, while the less ideologically aligned adopt it as their primary channel of participation when voting seems insufficient.

### 1.2. Study Context

On 18 October 2019, a fare increase on Santiago’s Metro system triggered an unprecedented social mobilization in Chile, the largest since the country’s return to democracy in 1990 (Scherman & Rivera, 2021; Giacoman & Torres, 2021). Just one week later, on 25 October 2019, approximately three million people—roughly 16% of the population—took to the streets without centralized leadership or formal party affiliation (Luna et al., 2022). These protests reflected deep-seated discontent over high levels of inequality, low trust in political elites and institutions, and a socio-economic system perceived as incapable of addressing demands for health, education, and pension reform (Scherman et al., 2021; Luna et al., 2022; Torres, 2022).

During the 2019 demonstrations, young Chileans leveraged social media to broadcast protest calls, document state abuses, and disseminate firsthand testimonies, functioning as a horizontal channel to amplify their demands (Scherman et al., 2021; Giacoman & Torres, 2021). Prior research indicates that social media not only facilitated logistical coordination and real-time information exchange but also, in many cases, supplanted traditional organizational practices of social movements (Torres & Costa, 2012; Valenzuela et al., 2016). Indeed, during the uprising, 50% of Chilean youth reported sharing protest-related content on social networks, and they relied on WhatsApp (51%), Facebook (42%), Instagram (38%), and Twitter (13%) as their primary sources of information and mobilization (UDP Feedback, 2019). These figures underscore both the central role of these platforms and young people’s high dependence on them for news and collective action during the protests (Scherman & Rivera, 2021).

The mass mobilizations culminated in the launch of Chile’s constituent process. Following the “Agreement for Social Peace and a New Constitution” in November 2019, a plebiscite was scheduled for April 2020 (postponed to October due to the COVID-19 pandemic), and in May 2021, delegates to the Constitutional Convention were elected (Torres et al., 2023; Negretto, 2021). Throughout this period, social media remained central—especially among young Chileans—with over 3.5 million Twitter posts related to the Constitutional Process, peaking around key events such as the signing of the Agreement and the campaign for the plebiscite (Ruiz-Dodobara et al., 2025; Durán et al., 2020). Yet, the period also revealed challenges: more than 50% of those who followed the Convention via social media reported encountering misinformation, and 66% of circulating content originated on Facebook, Twitter, or Instagram (Datavoz, 2022).

These events demonstrate that the October 2019 protests and the onset of the Constituent Process in 2021 not only reactivated youth political interest (Torres et al., 2023; INJUV, 2022; Torres, 2024) but also strengthened the perception of social media as legitimate channels for participation. Indeed, a 2022 survey by the National Youth Institute (INJUV) found that 45% of young Chileans during the post-Uprising and constitutional phase considered social media a more effective tool than voting for expressing popular demands. This percentage is the highest recorded in recent years (INJUV, 2022).

### 1.3. Aim of This Study

In summary, existing research indicates that political interest, democratic satisfaction, and political identification shape how Chilean youth may perceive social media as a superior tool to voting for expressing social demands, particularly in highly politicized contexts. However, important gaps remain. First, much of the literature relies on cross-sectional data, underscoring the need for longitudinal studies that compare youth perceptions of social media before and after major sociopolitical events (Eckstein et al., 2024). Second, there is a paucity of research comparing these perceptions specifically between adolescents and young adults in relation to social media versus voting.

This article addresses these gaps through three objectives: (1) to assess whether, following the 2019 Social Uprising and up to the start of the Constitutional Process in May 2021, there has been a statistically significant increase in favorable attitudes toward social media as a more effective tool than voting for expressing social demands; (2) to examine how these attitudes toward social media versus voting vary among adolescents (15–17) and young adults (18–21 and 22–24); and (3) to analyze the role of political interest, satisfaction with democracy, and political identification as predictors of attitudes toward social media versus voting.

## 2. Materials and Methods

### 2.1. Data Used

This quantitative study draws on data from the National Youth Survey (NYS) [Encuesta Nacional de Juventudes, ENJ], conducted by the Chilean National Youth Institute (INJUV). The NYS is recognized as one of the most robust and technically consistent instruments in Ibero-America for profiling the population aged 15–29 years (INJUV, 2022). It is a nationally, regionally, and urban–rural representative survey based on a stratified probabilistic sampling design. For the present analysis, we use data from the 9th NYS, fielded between December 2018 and April 2019, and the 10th NYS, carried out from December 2021 to May 2022.

### 2.2. Sample

For this study, the analytic sample includes adolescents and young adults aged 15–24 years. The 9th NYS comprised 6198 respondents (mean age = 20.0 years, SD = 2.69), of whom 48.9% were male and 51.1% were female. The 10th NYS comprised 6437 respondents (mean age = 19.0 years, SD = 2.81), with 47.6% male and 52.4% female. Table 1 further details the age distribution across three groups: 15–17, 18–21, and 22–24 years.

### 2.3. Variables and Measures

The dependent variable captures respondents’ level of agreement with the statement that social media is a better tool than voting for expressing civic demands. It was measured via the survey item asking respondents to rate, on a scale from 1 (‘strongly disagree’) to 5 (‘strongly agree’), the following statement: *Social media is a better tool than voting for making people’s demands known*. Higher scores indicate a stronger preference for social media over voting as a mechanism of political expression.

Based on our literature review, three sociopolitical attitudes were selected as variables associated with perceiving social media as a more effective tool than voting for expressing social demands. *Political interest* was measured with the question, “How interested are you in politics?” on a 4-point scale (1 = not at all interested; 4 = very interested). *Satisfaction with democracy* was assessed by asking, “How satisfied are you with democracy in Chile?” on a 5-point scale (1 = very dissatisfied; 5 = very satisfied). *Political identification* was captured with the item, “With which political sector do you feel most identified?” offering six response options: Right, Centre-Right, Centre, Centre-Left, Left, and None of these. Due to low frequencies in each category, all ideological positions were combined into a single “identified” group (see Table 1). For analysis, this variable was then dichotomized as 1 = Identified (any declared ideological stance) and 0 = Non-identified.

To compare political attitudes toward social media across developmental stages, age was categorized into three groups based on legal status and psychosocial development. The first group comprises adolescents aged 15–17, who have not yet reached the legal age of majority nor obtained suffrage rights in Chile. The second group includes emerging adults aged 18–21, who are legally enfranchised but remain in the transitional phase of emerging adulthood. The final group consists of young adults aged 22–24, an age range that multiple studies identify as the gradual culmination of the transition to full adulthood, marked by higher levels of personal, occupational, and civic autonomy. This classification enables capturing meaningful differences in participatory experiences across distinct stages of the youth life course (Maurissen & Claes, 2023; Oden & Porter, 2023).

In addition to the main predictors, we included key sociodemographic controls shown in prior research to influence political attitudes. Specifically, we accounted for gender (0 = male; 1 = female) and socioeconomic status (SES). SES was estimated based on respondents’ self-reports, place of residence, and interviewers’ assessments, classifying individuals into five strata (ABC1, C2, C3, D, and E, from highest to lowest). For analysis, SES was recoded into a five-point ordinal scale ranging from lowest to highest socioeconomic level.

### 2.4. Analytical Approach

To address our study objectives, we employed a three-step analytical strategy. First, independent-samples *t*-tests and one-way analyses of variance (ANOVAs) were conducted to detect significant differences in the dependent variable across age groups (15–17, 18–21, and 22–24 years) in both survey waves, following established practices in political behavior research involving age-group comparisons. Second, Pearson correlation matrices were computed between the dependent variable and each independent variable (political interest, democratic satisfaction, and political identification) to assess bivariate associations in line with conventional correlational research practices in the social sciences. Finally, multiple linear regression analyses were performed: Model 1 included only the sociopolitical predictors; Model 2 added age groups as control variables; and Model 3 further incorporated all sociodemographic controls (gender and socioeconomic status). This approach allows us to evaluate the robustness of sociopolitical predictors while accounting for relevant individual characteristics, a methodological strategy widely employed in contemporary research on political behavior and social media (Maurissen & Claes, 2023; Ruiz-Dodobara et al., 2025). Descriptive and bivariate analyses were conducted in SPSS v. 27, and multivariate regressions were estimated in Jamovi v. 2.3.28.0.

## 3. Results

Data from the 9th and 10th National Youth Surveys (2018–2019 and 2021–2022, respectively) reveal a clear shift in adolescents’ and young adults’ attitudes toward social media as a channel for civic expression compared to voting. Specifically, there is a widespread increase in the endorsement of these platforms as more effective than voting for channeling social demands. This attitudinal change temporally coincides with the October 2019 Social Uprising and the subsequent Constitutional Process, suggesting that context played a role in shaping youth political participation. The analyses presented below disaggregate results by age group, compare both survey periods, and examine the sociopolitical factors associated with these attitudinal shifts.

### 3.1. Age-Group Variations in Attitudes Toward Social Media Versus Voting Across Periods

As shown in Table 2, there is a marked increase in attitudes favoring social media over voting as a tool for expressing civic demands between the 9th and 10th National Youth Surveys, both for the overall sample and across each age group. Although all age groups exhibited significant gains, the largest shift occurred among adolescents aged 15–17, whose mean attitude score rose from 2.95 to 3.45 over the two periods—a change of +0.50.

Independent-samples *t*-tests comparing mean scores for social media attitudes between surveys revealed statistically significant increases for the total sample and for each age group (all *p* < 0.001). Cohen’s d effect sizes indicate medium effects overall (d = 0.32–0.44), with the strongest effect among adolescents (d = 0.44), followed by emerging adults aged 18–21 (d = 0.39) and young adults aged 22–24 (d = 0.32). These findings indicate that support for social media as a channel for civic expression increased in a statistically significant manner from the pre-Uprising period (2018–2019) to the post-Uprising/Constitutional period (2021–2022).

**Figure 1:**
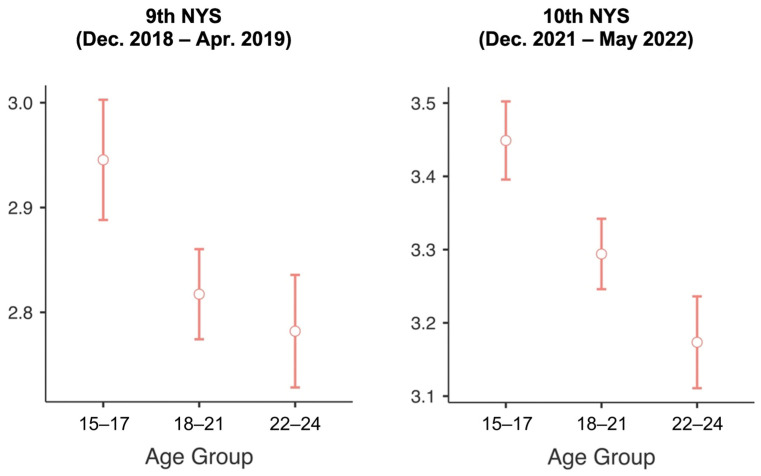
Mean Attitude Scores Toward the Statement “Social Media Are More Effective Than Voting for Expressing Citizens’ Demands,” by Age Group and Period (95% CI).

Figure 1 presents estimated means and 95% confidence intervals for the attitude statement “Social media are a better tool than voting for making people’s demands known” across age groups for December 2018–April 2019 and December 2021–May 2022.

For December 2018–April 2019, the 15–17 age group showed the highest agreement (M = 2.95, SD = 1.09), followed by 18–21 (M = 2.82, SD = 1.10) and 22–24 (M = 2.78, SD = 1.12). A one-way ANOVA confirmed significant differences among age groups (F(2, 5588) = 9.20, *p* < 0.001). Post hoc Games-Howell tests revealed that adolescents aged 15–17 differed significantly from those aged 18–21 (Δ = 0.13; *p* = 0.001) and 22–24 (Δ = 0.16; *p* < 0.001), whereas no significant difference emerged between the two older groups (*p* = 0.573). These findings suggest that, prior to the Social Uprising, younger adolescents valued social media as a channel of expression more highly than young adults.

For December 2021–May 2022, the 15–17 age group again registered the highest level of agreement (M = 3.45, SD = 1.19), followed by 18–21 (M = 3.29, SD = 1.27) and 22–24 (M = 3.17, SD = 1.33). A one-way ANOVA confirmed significant differences among age groups (F(2, 6347) = 21.88, *p* < 0.001). Post hoc comparisons revealed significant differences for all pairwise contrasts: 15–17 vs. 18–21 (Δ = 0.16; *p* < 0.001), 15–17 vs. 22–24 (Δ = 0.28; *p* < 0.001), and 18–21 vs. 22–24 (Δ = 0.12; *p* = 0.008). These findings indicate that, beyond the overall increase in endorsement of social media as a tool for civic expression following the Social Uprising and during the Constitutional Process, adolescents (15–17) consistently held more favorable attitudes than young adults, and that in the post-Uprising period, emerging adults (18–21) also rated social media significantly higher than the 22–24 age group.

### 3.2. Sociopolitical Predictors of Attitudes Toward Social Media Versus Voting

To assess the role of sociopolitical variables in the attitude that social media are more effective than voting for expressing civic demands, six linear regression models were estimated (Table 3). Models M1–M3 correspond to the 9th NYS (December 2018–April 2019) and Models M4–M6 to the 10th NYS (December 2021–May 2022). Models M1 and M4 include only the three core predictors; Models M2 and M5 add age group controls; and Models M3 and M6 further include gender and socioeconomic status.

Regarding political interest, in the 9th NYS (December 2018–April 2019), this variable showed no statistically significant association with the perception of social media as more effective than voting, even after controlling for age, gender, and SES (Model M3: β = −0.02; *p* > 0.05). This suggests that, prior to the Social Uprising, young people’s level of political interest did not influence their valuation of digital platforms. However, in the 10th NYS (December 2021–May 2022), political interest was robustly and negatively associated with this attitude (Model M6: β = −0.12; *p* < 0.001), indicating that, following the Constitutional Process, youths with higher political interest tend to perceive social media as less effective compared to voting, whereas those with lower political interest exhibit the opposite pattern, assigning greater effectiveness to social media over voting.

Satisfaction with Democracy emerged as a positive and significant predictor in both periods. In the 9th NYS, this effect remained robust after controlling for all sociodemographic variables (M3: β = 0.09; *p* < 0.001), indicating that respondents who are more satisfied with democracy also rate social media more highly as a channel for political expression. In the 10th NYS, although the coefficient’s magnitude decreased slightly (M6: β = 0.06; *p* < 0.001), the relationship remained statistically significant, suggesting a stable link between trust in democratic institutions and the perception of social media as legitimate spaces for participation.

Political Identification—coded 1 for those reporting any ideological affiliation and 0 for those without—showed a marked shift between periods. In the first period (M3), its negative association with social media valuation was not statistically significant (β = −0.07; *p* > 0.05), implying that, before the Social Uprising, declaring an ideological stance did not affect digital attitudes. In the second period (M6), this relationship became both significant and stronger (β = −0.12; *p* < 0.001), indicating that unaffiliated youth assign greater effectiveness to social media for channeling civic demands, which may reflect limited confidence in parties and traditional political structures.

Finally, when sociodemographic controls were added in Models M3 (2018–2019) and M6 (2021–2022), several notable nuances emerged. In both periods, adolescents aged 15–17 continued to exhibit the highest endorsement of social media as a political tool, whereas the 18–21 and 22–24 age groups showed significant negative coefficients that grew more pronounced in 2021–2022 (M6: β = −0.11 and β = −0.24, respectively; *p* < 0.001). Additionally, in the post-Uprising context, gender and socioeconomic status became relevant predictors: females assigned significantly greater value to the political role of social media over voting (M6: β = 0.16; *p* < 0.001), while those from higher socioeconomic strata perceived social media as less effective (M6: β = −0.08; *p* < 0.001).

**Figure 2:**
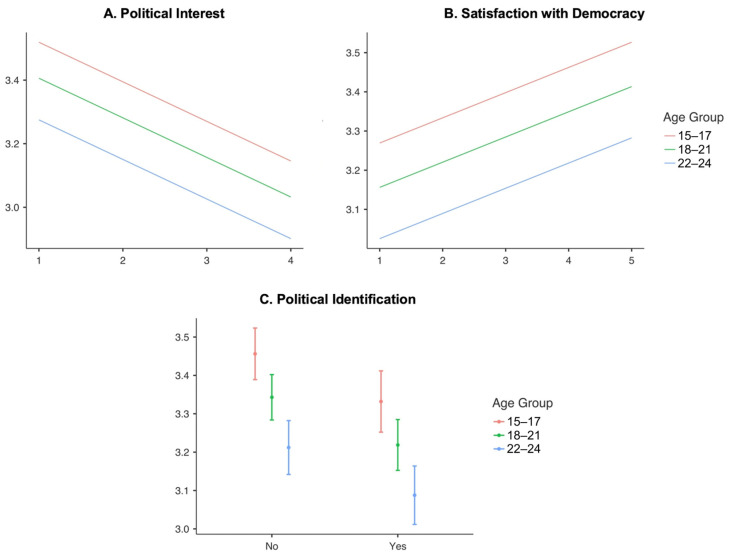
Estimated Marginal Means of Attitudes Toward the Statement “Social Media Are More Effective Than Voting for Expressing Citizens’ Demands,” by Political Predictors and Age Group.

Figure 2 shows the estimated marginal means for predicted attitudes toward the statement “Social media are more effective than voting to express citizens’ demands,” based on the three sociopolitical predictors included in Model 6, disaggregated by age group. As shown in Figure 2A, support for social media as a more effective channel of civic expression than voting decreases as political interest increases across all age groups. Although this downward trend is consistent among adolescents (15–17), emerging adults (18–21), and young adults (22–24), the highest levels of support are observed among the youngest cohort, followed by those aged 18–21, with the lowest levels among respondents aged 22–24.

In Figure 2B, satisfaction with democracy reveals an opposite pattern: higher levels of satisfaction are associated with stronger agreement that social media are more effective than voting for expressing citizens’ demands. This positive association is consistent across all three age groups, with adolescents (15–17) showing the most pronounced increase in support, followed by those aged 18–21 and, to a lesser extent, young adults aged 22–24.

Figure 2C highlights the effect of political identification. In all age groups, respondents who do not identify with any political ideology express greater agreement that social media are more effective than voting for articulating citizens’ demands, compared to those who do report a political identification. As with the previous predictors, this pattern is most pronounced among adolescents aged 15–17.

These findings show that higher political interest and political identification are associated with weaker agreement that social media are more effective than voting for expressing civic demands, whereas lower political interest and lack of political identification correspond to stronger endorsement of social media’s effectiveness. Moreover, greater satisfaction with democracy consistently strengthens this perception. This pattern holds across all age groups.

## 4. Discussion

In this study, we examined whether and how the attitudes of Chilean adolescents and young adults toward social media—as a more effective tool than voting for expressing social demands—changed in the wake of the October 2019 Social Uprising and the onset of the Constitutional Process. To do so, we compared two periods: Pre-Social Uprising (December 2018–April 2019) versus Post-Social Uprising (December 2021–May 2022). We also explored age-related differences across three developmental stages (15–17, 18–21, and 22–24 years old), and analyzed the role of political interest, satisfaction with democracy, and political identification in shaping these attitudes.

Our findings reveal a statistically significant increase in support for social media over voting across all youth groups after the 2019 uprising, with the most pronounced rise observed among adolescents (15–17 years old). Regression analyses further show that, following the 2019 Social Uprising, political interest shifted from being a non-significant predictor to a significantly negative one. A similar shift occurred with political identification, which also became a significantly negative predictor. In contrast, satisfaction with democracy remained a stable and positive predictor across both periods. Marginal means analyses confirmed these patterns across all age groups.

Regarding the results, the overall upward trend in youth attitudes toward social media aligns with broader reconfigurations of political participation triggered by social and political crises, as documented in studies emerging since the global wave of protests in the 2010s (Bennett & Segerberg, 2012; Valenzuela et al., 2016; Torres & Costa, 2012). In the Chilean case, the October 2019 Uprising—and the increasing politicization surrounding the Constitutional Process—appear to have accelerated adolescents’ and young people’s valuation of digital platforms as viable spaces for civic expression, complementing or even replacing traditional electoral mechanisms (Fierro et al., 2023; Espinoza Bianchini et al., 2022; Scherman & Rivera, 2021; Torres, 2024). This resonates with research on collective action in digitally mediated contexts, where moments of intense sociopolitical disruption amplify the perceived instrumental value of online networks (Waeterloos et al., 2024; Ruiz-Dodobara et al., 2025; Valenzuela et al., 2016; Theocharis et al., 2014).

Continuing with the findings, age-group differences highlight that adolescents (15–17) consistently held more favorable attitudes toward social media versus voting than emerging adults (18–21) and young adults (22–24). The most marked attitudinal shift among the youngest group may reflect both their developmental stage—characterized by higher digital fluency and peer-centered socialization—and their weaker ties to institutional forms of participation, not least because they have not yet reached the legal voting age in Chile (Maurissen & Claes, 2023; Benavente et al., 2023). This finding highlights the relevance of distinguishing between adolescence and young adulthood in models of digital engagement by demonstrating that the political implications of digital participation vary even within the broader “youth” category (Oden & Porter, 2023).

Regarding the sociopolitical predictors, satisfaction with democracy emerged as a significant and positive predictor in both periods, suggesting that, both before and after the 2019 uprising, higher levels of democratic satisfaction were associated with the adoption of social media as a channel for civic expression. This finding aligns with certain strands of international research, which indicate that in countries with strong and consolidated democracies, the political use of social media tends to be positively related to satisfaction with democracy (Placek, 2023; Chan & Yi, 2024).

In contrast, the significant negative association of political interest observed after 2019 suggests that adolescents and young people who are less politically engaged are more likely to value social media as an alternative to voting for expressing civic demands. Similarly, the negative effect of political identification indicates that those without political affiliation—possibly disenchanted with formal politics—are especially inclined to favor social media as an expressive outlet. Conversely, more politically engaged adolescents and youth may continue to trust voting more or view social media as less effective compared to their less politicized peers. These patterns were consistent across all age groups but were particularly pronounced among adolescents (15–17), who not only displayed the most favorable attitudes toward social media but also experienced the sharpest post-Uprising shifts.

Taken together, these findings provide additional insight into trends identified in the literature, which typically associate higher levels of political interest or ideological positioning with greater political participation or political use of social media among adolescents and young people (Maurissen & Claes, 2023; Lorenz-Spreen et al., 2023; Oden & Porter, 2023; Espinoza Bianchini et al., 2022; Fierro et al., 2023). Our results reveal that lower levels of political interest and identification may be linked to a greater valuation of online participation relative to voting. At the same time, they reinforce previous studies showing that low political interest may be associated with increased use of social media as a means of expression and discussion (Bimber et al., 2014).

While this study offers valuable longitudinal evidence on shifts in attitudes toward social media versus voting in contexts of political crisis, it is important to acknowledge its limitations and suggest directions for future research. Although the data from the National Youth Survey (NYS) are nationally representative, the survey relies on repeated cross-sectional designs rather than a longitudinal panel study, which entails important methodological differences. Moreover, the instrument did not differentiate between specific platforms (e.g., TikTok, Instagram, etc.), which may be particularly relevant given the distinct preferences of different age groups. Future research could benefit from the implementation of panel designs with more frequent measurements, allowing for a more precise understanding of causal dynamics. At the same time, combining quantitative and qualitative methods—such as in-depth interviews and digital ethnography—would help uncover the motivational processes underlying the political use of social media. Additionally, extending the analysis to comparative contexts, including other Latin American or European countries, could offer valuable insights into how sociopolitical variables interact with institutional and cultural factors in shaping the perceived civic role of social media.

## 5. Conclusions

This study shows that Chilean youth attitudes toward social media—compared to voting—have shifted following key political events. Comparing the periods before and after the Social Uprising (December 2018–April 2019 vs. December 2021–May 2022), there was an increase in the belief that social media offers a better alternative than voting for expressing citizens’ demands, particularly among adolescents aged 15–17. In the period following the October 2019 Social Uprising and the beginning of the Constitutional Process, greater political interest and stronger political identification were associated with a lower valuation of social media as an alternative to voting. Conversely, higher levels of satisfaction with democracy were linked to a greater appreciation of social media compared to voting. More defined social and political patterns also emerged during this period: younger adolescents with low political interest and identification, and who belong to middle- or lower-income groups, were the most likely to value social media over voting as a channel for expressing demands. These findings highlight how youths’ attitudes toward digital platforms are deeply intertwined with political engagement and socioeconomic background. They also carry important implications for policymakers and civil society actors, who should recognize these dynamics when designing initiatives in civic education, voting promotion, and digital participation. Finally, this research contributes to a broader understanding of how young people are reshaping the boundaries of political participation in the digital era and highlights the need for further studies on the long-term consequences of this transformation.

## Figures and Tables

**Table 1:** Descriptive statistics.

Variable	9th NYS (December 2018–April 2019)	10th NYS (December 2021–May 2022)
Agreement Level to Social Media as a Better Tool than Voting	2.84 (1.11)	3.31 (1.27)
Gender		
Female	3170 (51.1%)	3372 (52.4%)
Male	3028 (48.9%)	3065 (47.6%)
Age Group		
15–17	1611 (26.0%)	1958 (30.4%)
18–21	2764 (44.6%)	2733 (42.5%)
22–24	1823 (29.4%)	1746 (27.1%)
Socio-economic group	2.86 (1.01)	2.80 (0.92)
Political Interest	1.60 (0.83)	1.96 (0.92)
Satisfaction with Democracy	2.67 (0.94)	2.94 (0.98)
Political identification		
Yes	1479 (23.9%)	2243 (36.8%)
No	4180 (67.4%)	3845 (63.2%)
Complete obs.	6198	6437

*Note*: Frequencies and percentages for categorical variables; mean and SD for continuous ones.

**Table 2:** Changes in Attitudes Toward Social Media as a Tool for Expression Between 2018–2019 and 2021–2022, by Age Group.

Age	9th NYS December 2018–April 2019	10th NYS December 2021–May 2022	Mean Difference	t (df)	Cohen’s d
15–17	2.95 (1.09)	3.45 (1.19)	+0.50	7.12 (3300) ***	0.44
18–21	2.82 (1.10)	3.29 (1.27)	+0.47	8.05 (5200) ***	0.40
22–24	2.78 (1.12)	3.17 (1.33)	+0.39	5.95 (3400) ***	0.32
Total (15–24)	2.84 (1.11)	3.31 (1.27)	+0.47	11.30 (11,900) ***	0.41

*Note*: Dependent variable: Agreement with “Social media is a better tool than voting for expressing social demands”. Scores measured on a 1 (strongly disagree) to 5 (strongly agree) scale; standard deviations in parentheses. *** *p* < 0.001.

**Table 3:** Multi-level linear Regression Models.

	9th NYS Dec. 2018–Apr. 2019			10th NYS Dec. 2021–May 2022		
Predictor	Base Model (M1)	+Age Controls (M2)	+Full Controls (M3)	Base Model (M4)	+Age Controls (M5)	+Full Controls (M6)
Political interest	–0.02 (0.02)	–0.02 (0.02)	−0.02 (0.02)	−0.13 (0.02) ***	−0.13 (0.02) ***	−0.12 (0.02) ***
Satisfaction with democracy	0.10 (0.02) ***	0.09 (0.02) ***	0.09 (0.02) ***	0.07 (0.02) ***	0.06 (0.02)	0.06 (0.02) ***
Political identification (ref. No)	–0.06 (0.04)	–0.06 (0.04)	−0.07 (0.04)	−0.17 (0.04) ***	−0.15 (0.04) ***	−0.12 (0.04) ***
Age (ref. 15–17 years)		–			–	
18–21 years		−0.10 (0.04) *	−0.09 (0.04) *		−0.13 (0.04) **	−0.11 (0.04) **
22–24 years		−0.13 (0.04) **	−0.13 (0.04) **		−0.25 (0.04) ***	−0.24 (0.05) ***
Gender (ref. Male)			0.03 (0.03)			0.16 (0.03) ***
Socioeconomic group			0.02 (0.02)			−0.08 (0.02) ***
Constant	2.62 (0.06) ***	2.72 (0.07) ***	2.66 (0.08) ***	3.41 (0.06) ***	3.55 (0.07) ***	3.65 (0.09) ***
Complete obs.	6198	6198	6198	6437	6437	6437
R^2^	0.008	0.011	0.010	0.020	0.025	0.032

*Note:* Dependent variable: Agreement with “Social media is a better tool than voting for expressing social demands”. Scores measured on a 1 (strongly disagree) to 5 (strongly agree) scale. Unstandardized parameter estimates with standard errors in parentheses are reported. * *p* < 0.05; ** *p* < 0.01; *** *p* < 0.001.

## Data Availability

The data used in this study are publicly accessible and available at: https://www.injuv.gob.cl/encuestanacionaldejuventud (accessed on 2 July 2025).
